# Exploratory pharmacodynamics and efficacy of PF-06817024 in a Phase 1 study of patients with chronic rhinosinusitis and atopic dermatitis

**DOI:** 10.1186/s13223-024-00894-8

**Published:** 2024-08-30

**Authors:** Spencer I. Danto, Nikolaos Tsamandouras, Padma Reddy, Steven A. Gilbert, Jessica Y. Mancuso, Karen Page, Jean S. Beebe, Elena Peeva, Michael S. Vincent

**Affiliations:** Pfizer Inc, 1 Portland Street, Cambridge, MA 02151 USA

**Keywords:** Anti-IL-33 antibody, Chronic rhinosinusitis with nasal polyps, Atopic dermatitis, Phase 1, Efficacy, Biomarkers, Pharmacodynamics

## Abstract

PF-06817024 is a humanized antibody against interleukin-33 that has the potential to inhibit type 2 inflammation. An exploratory analysis of the pharmacodynamics and clinical effects of single and repeat doses of PF-06817024 was assessed in patients with chronic rhinosinusitis with nasal polyps (CRSwNP) and patients with moderate-to-severe atopic dermatitis (AD), respectively, as part of a Phase 1, first-in-human study. Rhinosinusitis symptoms were improved, and nasal polyps were decreased in size following treatment with PF-06817024 in patients with CRSwNP. In patients with AD, PF-06817024, in aggregate, reduced disease severity and improved symptoms, as demonstrated by greater percentage decrease from baseline in Eczema Area and Severity Index (EASI) scores and reduced pruritus numerical rating scores, compared with placebo. The efficacy in AD appeared to be bimodal with a sub-group of participants exhibiting high levels of improvement (EASI75 and EASI90) for a sustained period of time after dosing. In patients with CRSwNP, a consistent trend of decrease in eosinophil levels was observed in the PF-06817024 group, compared with placebo. Further research would be needed to confirm the clinical benefit and safety of PF-06817024 as a treatment for allergic diseases.

**Trial registration** ClinicalTrials.gov, NCT02743871. Registered 15 April 2016, https://clinicaltrials.gov/study/NCT02743871?term=NCT02743871&rank=1.

**Supplementary Information:**

The online version contains supplementary material available at 10.1186/s13223-024-00894-8.

## Introduction

Allergic diseases such as asthma, allergic rhinitis, and atopic dermatitis (AD) represent major public health challenges, with the global prevalence of such diseases increasing each year [1, 2]. Chronic rhinosinusitis with nasal polyps (CRSwNP) is an inflammatory disease with a reported prevalence ranging from 1.0 to 2.6% [3]. Common symptoms include sinus pressure, nasal congestion, and a decreased sense of smell [4], which cause patients with CRSwNP to have a lower quality of life [5]. AD is a chronic inflammatory skin disease that typically starts in infancy, with reported prevalence in children ranging from 2.7 to 20.1%; however, it is also highly prevalent in adults, with a range of 2.1–4.9% [6–8]. Clinical manifestations of AD vary with age, with the scalp, face, neck, and trunk affected in infants, while in children and adolescents the flexural surfaces of the extremities are typically more affected [9]. AD significantly impairs quality of life as the disease is associated with anxiety, depression, and sleep disturbances [10, 11].

Type 2 inflammation has been implicated in the pathophysiology of atopic diseases such as CRSwNP and AD. The type 2 inflammatory response is mediated, in part, by T helper 2 (T_H_2) cells and type 2 innate lymphoid cells (ILC2s) [12]. Following exposure to an allergen, damaged epithelial cells release alarmins, including interleukin (IL)-33 [13]. IL-33 binds to its receptor, suppression of tumorigenicity 2 (ST2), which is primarily located on mast cells and T_H_2 cells, and triggers the production of T_H_2 cytokines such as IL-4, IL-5, IL-13, and IL-31 [14, 15]. These cytokines contribute to the pathological hallmarks of allergic diseases; IL-4 and IL-13 are involved in immunoglobulin E (IgE) class switching in B cells, while IL-5 induces the production and survival of eosinophils [12]. In patients with moderate-to-severe AD, the onset of acute lesions was associated with significant increases in gene expression levels of IL-4 and IL-13 [16]. In addition, samples obtained from patients with CRSwNP also showed high levels of T_H_2 cytokines, including IL-4, IL-5, and IL-13 [17]. Since IL-33 drives the production of such cytokines, its blockade represents a promising therapeutic approach for both CRSwNP and AD. PF-06817024 is a humanized antibody against IL-33 that prevents IL-33 from binding to ST2, and thus triggering a type 2 inflammatory response that is characteristic of CRSwNP and AD.

This paper presents the results from an exploratory analysis of the pharmacodynamics (PD) and clinical effects of single or repeat doses of PF-06817024 in patients with CRSwNP and patients with moderate-to-severe AD, respectively, in the context of a first-in-human clinical study. The safety, tolerability, pharmacokinetics (PK), and immunogenicity of PF-06817024 in these populations were reported separately along with data from single and repeat doses in healthy volunteers.

## Methods

### Study design

The full study design is described in the companion publication of safety, tolerability, PK, and PD [18]. Briefly, the assessment of clinical effect and biomarkers of PF-06817024 were exploratory endpoints in a Phase 1, randomized, double-blind, placebo-controlled study that assessed the safety, tolerability, PK, and immunogenicity of PF-06817024 in healthy participants (Part 1), participants with CRSwNP (Part 2), and participants with AD (Part 3) (ClinicalTrials.gov, NCT02743871). The current brief report describes the results of exploratory analyses of signs of the clinical effect of PF-06817024 in participants with CRSwNP and AD. In Part 2 of the study, participants with CRSwNP were randomized (1:1) to receive a single intravenous (IV) dose of PF-06817024 300 mg or placebo. Participants were followed for > 211 days after the single dose, divided into a treatment period of 2 days, a follow-up period of 211 days, and an extended follow-up period thereafter, defined by the PK profile. In Part 3 of the study, participants with moderate-to-severe AD were randomized to receive repeat doses of PF-06817024 or placebo at a ratio of 2:1, although actual recruitment was closer to a ratio of 3:1, resulting in a mixed randomization ratio. The dosing regimen in Part 3 consisted of a single 600 mg IV loading dose, followed by three IV doses of 300 mg every 4 weeks. This dosing regimen was guided by emerging total IL-33 clinical data suggesting that higher and more frequent (monthly) dosing may be needed clinically than that predicted by in vitro antibody affinity assays for continuous, high level, suppression of IL-33 levels; a detailed rationale of the dosing regimens has been provided previously [18]. The defined treatment period was 113 days, with a standard follow-up period of 337 days, and an extended follow-up period thereafter. The extended follow-up period for both parts of the study was necessitated by the extended half-life of PF-06817024 (83–94 days) observed in Parts 1 and 2 of the study. For all parts of this study, blood samples were collected before dosing and at time points specific for the participant cohort as described previously [18]. All parts of this study were participant- and investigator-blinded.

The study was conducted in compliance with the ethical principles originating in or derived from the Declaration of Helsinki and in compliance with all International Council for Harmonisation of Good Clinical Practice Guidelines.

### Participants

#### CRSwNP

Participants aged 18–65 years with a body mass index (BMI) of 17.5–35 kg/m^2^ and a total body weight of > 50 kg with a history of CRSwNP were eligible for inclusion if they had: a minimum bilateral nasal polyp score (NPS) of 5 out of a maximum score of 8 and the presence of at least two of the following symptoms prior to screening: nasal blockade/obstruction/congestion, nasal discharge, facial pain/pressure, and reduction or loss of smell; were otherwise healthy (comorbid, controlled asthma with a forced expiratory volume in 1 s [FEV_1_] > 60% predicted was permitted). Participants were excluded from the study if they had received anti-IgE or anti-IL-5 therapy within 130 days prior to screening, had a 22-item Sino-nasal Outcome Test (SNOT-22) score < 7, or had undergone any nasal surgery within 6 months before screening.

#### AD

Participants aged between 18 and 75 years with a BMI of 17.5–40 kg/m^2^ and a total body weight of > 50 kg were eligible for inclusion if they had: a clinical diagnosis of chronic AD (for at least 1 year prior to Day 1) with an inadequate response to treatment with topical medications; moderate-to-severe AD, defined as having an affected body surface area (BSA) ≥ 10%, investigator global assessment (IGA) ≥ 3, and Eczema Area and Severity Index (EASI) score ≥ 12 at screening and baseline visits; were otherwise healthy (comorbid, controlled asthma with an FEV_1_ > 60% predicted was permitted). Participants were excluded if they had evidence of skin conditions such as psoriasis, seborrheic dermatitis, or lupus; had received systemic corticosteroids within 4 weeks prior to the first dose of the study drug; or had received dupilumab within 4 months of the first dose of the study drug or another anti-IL-4 and/or anti-IL-13 targeted therapies within 6 months of the first dose of the study drug.

### Endpoints

The pharmacologic effect of PF-06817024 was assessed by measuring the change from baseline in several serum biomarkers, including IL-5, IgE, chemokine (C-C motif) ligand 17 (CCL17), chemokine (C-C motif) ligand 26 (CCL26), circulating eosinophils, basophils, and ILC2s in participants with CRSwNP; and IgE, CCL17, and high-sensitivity C-reactive protein (hsCRP) in patients with AD.

In addition, exploratory measures of clinical effect of the single dose and multiple doses of PF-06817024 were also assessed in the CRSwNP and AD patient cohorts, respectively. In participants with CRSwNP, Lund-Mackay computerized axial tomography (CT) score [19], NPS [20], University of Pennsylvania Smell Identification Test (UPSIT) [21], the SNOT-22 score [22], and 5-item version of the Asthma Control Questionnaire (ACQ-5; asthmatic patients only) [23] were monitored. In participants with AD, the percentage change from baseline in the total EASI score, EASI50/75/90, affected BSA, IGA, and Scoring Atopic Dermatitis (SCORAD) were monitored. The change from baseline in patient-reported outcomes (PROs), including pruritus numerical rating scale, patient-oriented eczema measure, Dermatology Life Quality Index, Hospital and Anxiety Depression Scale, and ACQ-5 (asthmatic patients only) were also assessed.

### Statistical analyses

#### Statistical methods

All efficacy data were listed and summarized separately by part, and biomarker data were summarized descriptively. For Part 2 in patients with CRSwNP, as these were exploratory endpoints, no formal hypothesis testing was performed in this study. However, post-hoc efficacy and biomarker analyses were conducted, with least squares means (LSMs) and 80% confidence intervals (CIs) calculated based on analysis of covariance with independent variable of treatment groups and baseline result. Binary variables were summarized by the number of responders and the number of participants with data.

In Part 3, a mixed model for repeated measures (MMRM) analysis of change from baseline and percentage change from baseline in EASI scores was performed, with LSMs and 90% CIs presented for Day 113, the pre-specified landmark time point for assessment of clinical effect. The model included percentage change from baseline as the dependent variable and baseline EASI, treatment, study day, inverse study day (1/study day), treatment by study day, and treatment by inverse study day as fixed effects. Study day and inverse study day were included as continuous variables (not as class variables) and used the observed day (not the scheduled day). The MMRM analyses were performed in R using the lme package and included a random subject effect and a first-order autoregressive correlation over time. To understand the data generated by the pre-specified statistical analyses, additional post-hoc analyses were conducted.

## Results

### CRSwNP

In total, 20 participants with CRSwNP were randomized (11 in the PF-06817024 group and nine in the placebo group). A majority of randomized participants were male (65.0%), with a mean (standard deviation [SD]) age of 54.4 (6.2) years in the PF-06817024 group and 42.8 (10.7) years in the placebo group. Four participants with CRSwNP discontinued from the study: one participant in the PF-06817024 group discontinued during the extended follow-up period (Day 298); three participants in the placebo group discontinued during the extended follow-up period (> 211).

There was no consistent difference in change from baseline in Lund-Mackay CT score (mean [SD]: -5.1 [26.41]) in the PF-06817024 group, compared with placebo [(1.8 [31.43]). Generally, numerically greater decreases from baseline in NPS were observed during the follow-up period (Days 32–181) in the PF-06817024 group (-1.5 [1.92]) than in the placebo groups (-0.4 [1.13]). Increases from baseline in UPSIT scores were observed during the follow-up period (Days 61–211) in the PF-06817024 group (6.9 [10.14]) compared with decreases in the placebo group (-5.4 [11.41]), suggesting improved olfactory function in patients who received PF-06817024. Greater decreases from baseline in SNOT-22 scores were also observed in the PF-06817024 group (-13.5 [15.96]) compared with the placebo group (-5.7 [9.38]). In participants with comorbid asthma, lower ACQ-5 scores were observed in participants in the PF-06817024 group (-2.0 [2.83]) compared with participants in the placebo group (2.1 [7.20]) during the follow-up visits, suggesting improved asthma control. The clinical and PRO results suggest potential shrinkage of nasal polyps and modest improvement in symptoms and social and emotional consequences, respectively, and are suggestive of some beneficial effect of PF-06817024.

**Fig. 1:**
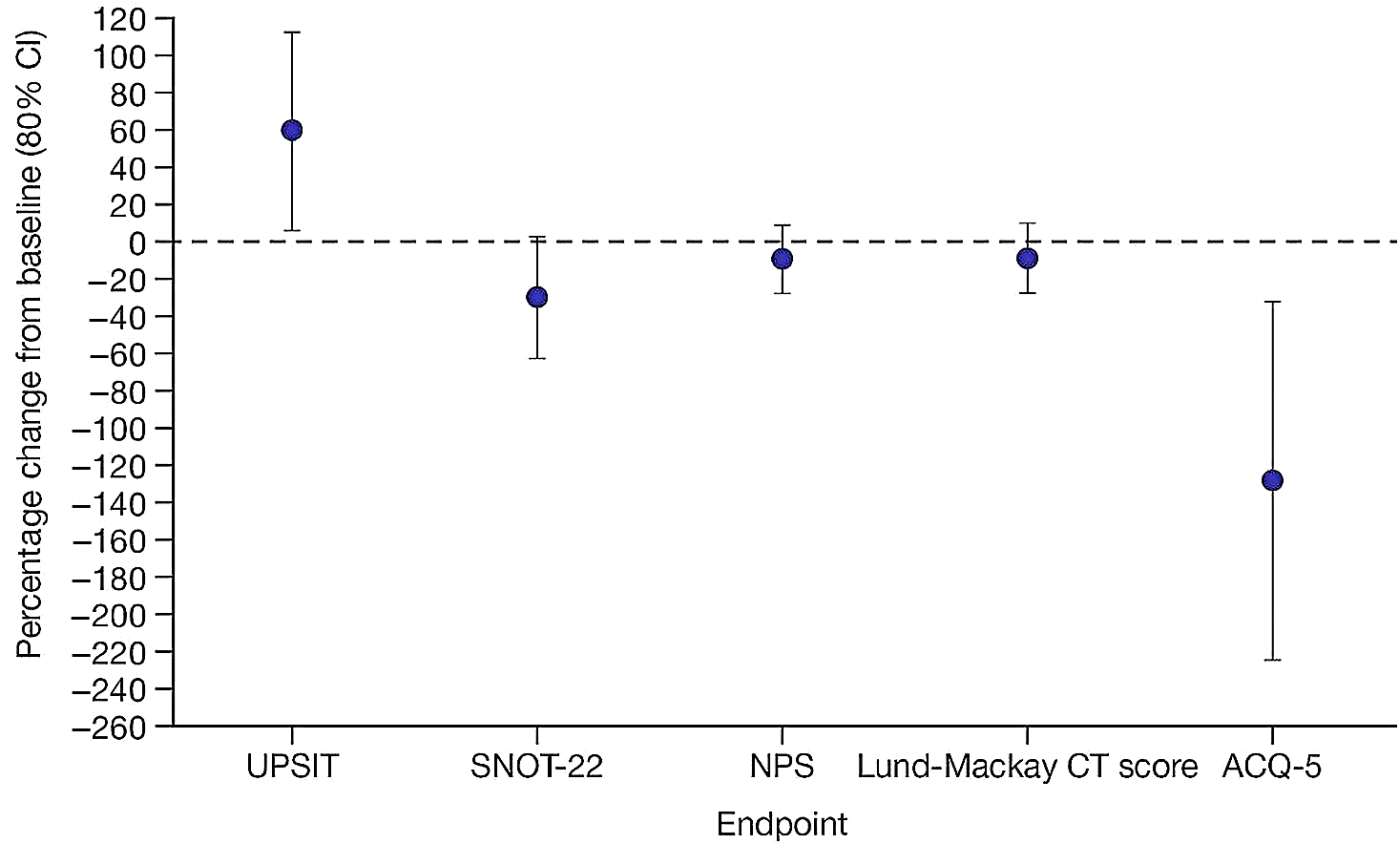
Percentage change from baseline in efficacy endpoints at Day 61 in patients with CRSwNP (placebo-corrected)^a^ Note: baseline is defined as the last measurement prior to first dosing. Circle represents LSM of percentage change from baseline in comparing with placebo. The estimate and CI were calculated based on ANCOVA analysis with independent variable of treatment groups and baseline result. Only asthmatic patients completed the ACQ-5. NPS data shown are for bilateral score. Lund-Mackay CT score was assessed by two expert reviewers; scores shown here are those from expert reviewer 1. Bars represent 80% CIs ^a^*n*=11 for UPSIT, SNOT-22, NPS, and Lund-Mackay CT score in the PF-06817024 group; *n* = 7 for ACQ-5 in the PF-06817024 group; *n* = 9 for UPSIT, SNOT-22, NPS, and Lund-Mackay CT score in the placebo group; and *n* = 7 for ACQ-5 in the placebo group ACQ-5, 5-item version of the Asthma Control Questionnaire; ANCOVA, analysis of covariance; CI, confidence interval; CRSwNP, chronic rhinosinusitis with nasal polyps; CT, computerized axial tomography; LSM, least squares mean; NPS, nasal polyp score; SNOT-22, 22-item Sino-nasal Outcome Test;UPSIT, University of Pennsylvania Smell Identification Test

For efficacy and PRO endpoints, data from Day 61 are shown in Table 1, and the percentage change from baseline in efficacy endpoints can be seen in Fig. 1.

**Table 1:** Effect of PF-06817024 on efficacy and patient-reported outcomes endpoints at Day 61 in patients with CRSwNP

	Baseline score		Change from baseline	
	PF-06817024 (*n* = 11)	Placebo (*n* = 9)	PF-06817024 (*n* = 11)	Placebo (*n* = 9)
Lund-Mackay CT score; mean (SD)^a, b^	15.1 (6.93)	15.4 (5.85)	-5.1% (26.41%)	1.8% (31.43%)
NPS; mean (SD)^b, c^	6.3 (0.79)	5.6 (0.73)	-1.5 (1.92)	-0.4 (1.13)
UPSIT; mean (SD)^c, d^	16.5 (10.58)	25.6 (11.95)	6.9 (10.14)	-5.4 (11.41)
SNOT-22; mean (SD)^b, c^	32.9 (17.60)	46.1 (16.19)	-13.5 (15.96)	-5.7 (9.38)
ACQ-5; mean (SD)^b, c, e^	2.9 (3.39)	2.9 (2.19)	-2.0 (2.83)	2.1 (7.20)

Note: Lund-Mackay CT score was assessed by two expert reviewers; scores shown here are those from expert reviewer 1. NPS data shown are for bilateral score

^a^Percentage change from baseline

^b^Decrease indicates improvement

^c^Numerical change from baseline

^d^Increase indicates improvement

^e^Only asthmatic patients completed the ACQ-5; *n* value was 7 for both the PF-06817024 and placebo groups at Day 61

ACQ-5, 5-item version of the Asthma Control Questionnaire; CRSwNP, chronic rhinosinusitis with nasal polyps; CT, computerized axial tomography; NPS, nasal polyp score; SD, standard deviation; SNOT-22, 22-item Sino-nasal Outcome Test; UPSIT, University of Pennsylvania Smell Identification Test

Decreases from baseline in blood eosinophil levels were observed at Day 61 with clear separation between PF-06817024 and placebo. However, there were no clear differences from placebo in other circulating biomarkers such as IL-5, IgE, CCL17, CCL26, ILC2s, and basophils at Day 61. LSMs, and associated 80% CIs, of percentage change from baseline for biomarkers at Day 61 are presented in Fig. S1A.

### AD

Overall, 28 patients with AD were randomized and treated (20 in the PF-06817024 group and eight in the placebo group). The randomized patients had a mean (SD) age of 38.9 (13.8) years in the PF-06817024 group and 41.0 (17.4) years in the placebo group, and 32.1% were male. There were 22 patients who discontinued: four in the PF-06817024 group and three in the placebo group during the treatment period; two patients each in the PF-06817024 and placebo groups during follow-up; nine in the PF-06817024 group and two in the placebo group during the extended follow-up period. The most common reason for discontinuation was “no longer willing to participate.”

**Fig. 2:**
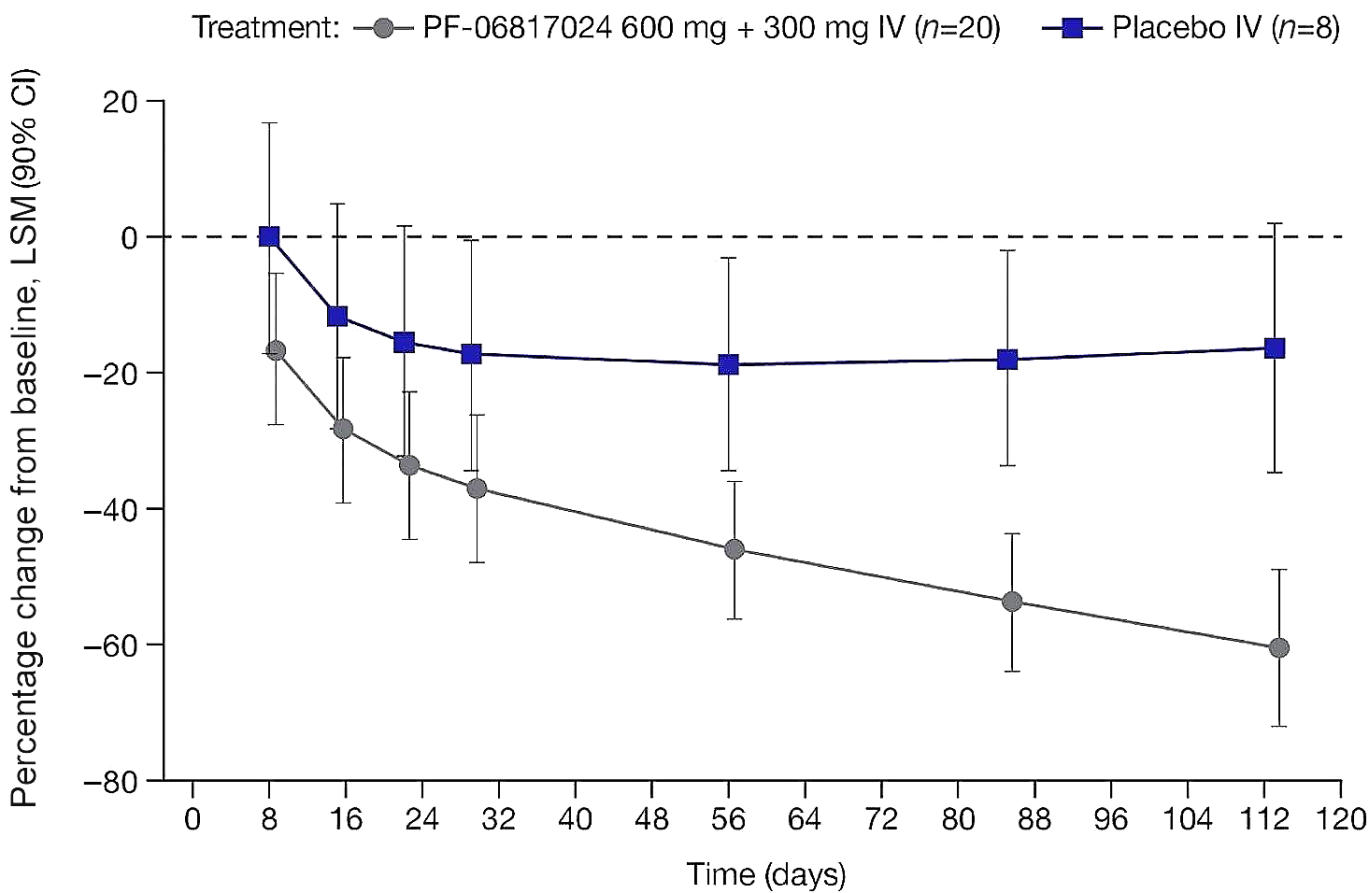
Longitudinal percentage change from baseline in EASI scores in patients with AD Note: baseline is defined as the last measurement prior to the first dosing. MMRM contains fixed factors of baseline EASI, treatment, study day, inverse study day, treatment by study day, and treatment by inverse study day and random factor of subject AD, atopic dermatitis; CI, confidence interval; EASI, Eczema Area and Severity Index; IV, intravenous; LSM, least squares mean; MMRM, mixed model repeated measures

A greater percentage decrease from baseline in total continuous mean EASI scores was observed in the PF-06817024 group, compared with placebo at Day 113, the pre-specified time point for assessment of clinical effect (Fig. 2). The LSM (90% CI) percentage change from baseline in EASI scores was − 60.4 (-71.9, -48.9) and − 16.2 (-34.5, 2.1) in the PF-06817024 and placebo group, respectively, at Day 113 (Table 2). In addition, a higher proportion of patients achieved a 50%, 75%, and 90% reduction in EASI score in the PF-06817024 group compared with the placebo group (Table 2), with responses observed up to Day 113. EASI scores continued to improve during the follow-up period (up to Day 337) and for a subset of the participants that remained in the study, a robust response was maintained even during extended follow-up (beyond Day 337; Fig. S2). Interestingly, post-hoc analyses of the individual responses indicated that the mean effect reflected the average of a bimodal/variable response pattern in which some patients experienced no response following administration of PF-06817024 at all timepoints, while others demonstrated high levels of improvement (75–90% EASI responses) (Fig. S3).

There were no clear separations from placebo observed in IgE, CCL17, and hsCRP in patients with AD. LSM (and associated 80% CIs) data of percentage change from baseline for biomarkers at Day 113 are presented in Figure S1B.

**Table 2:** Effect of PF-06817024 on EASI scores at Day 113 in patients with AD

	Baseline		Change from baseline	
	PF-06817024 (*n* = 20)	Placebo (*n* = 8)	PF-06817024 (*n* = 20)	Placebo (*n* = 8)
EASI^a^	20.9 (9.49)^b^	27.1 (17.47)^b^	-60.4 (-71.9, -48.9)^ c, d^	-16.2 (-34.5, 2.1)^ c, d^
EASI50; *n* (%)^e, f^	0 (0.0)	0 (0.0)	8 (57.1)	1 (20.0)
EASI75; *n* (%)^e, f^	0 (0.0)	0 (0.0)	6 (42.9)	0 (0.0)
EASI90; *n* (%)^e, f^	0 (0.0)	0 (0.0)	4 (28.6)	0 (0.0)

Note: baseline is defined as the last measurement prior to the first dosing. MMRM contains fixed factors of baseline EASI, treatment, study day, inverse study day, treatment by study day, and treatment by inverse study day

^a^*n*=21 for EASI in the PF-06817024 group; *n* = 7 for EASI in the placebo group

^b^Mean (SD)

^c^LSM (90% CI), percentage change from baseline

^d^Decrease indicates improvement

^e^Proportion of participants

^f^*n*=14 for EASI50, EASI75, and EASI90 in the PF-06817024 group; *n* = 5 for EASI50, EASI-5, and EASI90 in the placebo group

AD, atopic dermatitis; CI, confidence interval; EASI, Eczema Area and Severity Index; EASI50, ≥ 50% improvement from baseline in Eczema Area and Severity Index; EASI75, ≥ 75% improvement from baseline in Eczema Area and Severity Index; EASI90, ≥ 90% improvement from baseline in Eczema Area and Severity Index; LSM, least squares mean; MMRM, mixed model for repeated measures

## Discussion

Signs of clinical effect of PF-06817024 in patients with CRSwNP and AD were assessed as exploratory endpoints in a Phase 1, placebo-controlled, first-in-human study. Treatment with PF-06817024 led to a consistent improvement of symptoms in Part 2 of the study, while patients with AD also reported reduced disease severity, compared with those who received placebo.

IL-33 plays an important role in amplifying type 2 immune responses via its action on multiple target cells, including basophils, eosinophils, and ILC2s [24], and induction of cytokines such as IL-4, IL-5, and IL-13 [25, 26]. In patients with CRSwNP, IL-5, IgE, CCL17, CCL26, ILC2s, and circulating eosinophils and basophils were measured, while in patients with AD, IgE, CCL17, and hsCRP were monitored to assess any PF-06817024-mediated effect on IL-33 downstream activities. However, one limitation of this study was that biomarkers, such as cytokines and ILC2s, were not assessed in the more disease-relevant mucosal tissues where treatment-related changes may have been more prominent. A single dose of PF-06817024 in patients with CRSwNP was associated with reductions in circulating eosinophils, a biomarker that reflects circulating IL-5 activity [27]. Similar reductions in eosinophils were reported in a previous Phase 1 study with the IL-33 inhibitor itepekimab [28]. No consistent trends were observed in IL-5, IgE, CCL17, CCL26, ILC2s, or circulating basophils in patients with CRSwNP, or IgE, CCL17, and hsCRP in patients with AD. This may indicate that the type 2 inflammatory response may not have been fully inhibited by PF-06817024 either due to insufficient IL-33 neutralization or by the involvement of alternative pathways inducing type 2 inflammation. This could include other alarmins such as IL-25 that also promote the production of cytokines such as IL-4, IL-5, and IL-13, but are not inhibited by PF-06817024 [29, 30]. Considering the role of IL-33 and the alarmin thymic stromal lymphopoietin in activating the type 2 immune response [31], a combined blockade of both cytokines may be beneficial in patients with immunological conditions such as asthma or CRSwNP.

Total IL-33 levels were measured in the study as a surrogate of target engagement, and they appeared to have reached their maximum level/plateau by Day 61 in Part 2 (patients with CRSwNP) and Day 113 in Part 3 (patients with AD). These findings further support the a priori selection of these time points for assessment of exploratory efficacy and biomarker endpoints in these patient populations [18].

PF-06817024 had an effect on clinical outcome measures of CRSwNP severity, including improvement in symptoms of rhinosinusitis (USPIT and SNOT-22 scores), reduction in nasal polyp size and degree of nasal obstruction, and, in patients with comorbid asthma, improved asthma control (ACQ-5).

PF-06817024 demonstrated efficacy in patients with AD, as indicated by mean reductions in disease severity (EASI scores, IGA, BSA, and SCORAD) and patient-reported symptoms (pruritus and ACQ-5). Interestingly, the effects on the mean percentage change from baseline in EASI appeared to be reflective of dichotomous responses in two populations of patients: a responder population with high levels of improvement and a non-responder population who experienced placebo-like effects following administration of PF-06817024. No baseline characteristics could be identified that differentiated responders from non-responders (data not shown), which makes it difficult to predict who will benefit most from treatment with PF-06817024 in patients with AD. The modest aggregate clinical effects seen in the current study with PF-06817024 are consistent with results from clinical trials with other IL-33 inhibitors such as itepekimab and etokimab; however, these trials were conducted in patients with asthma and AD, respectively [28, 32]. It is not clear if similar bimodal responses have been seen with these other investigational products.

The assessments of PD and signs of clinical efficacy in patients with CRSwNP and AD described herein were done in the context of a dose-escalation, Phase 1 study to assess the safety, tolerability, PK, and immunogenicity of PF-06817024, principally conducted in healthy participants. The exploratory data with PF-06817024 in patients with CRSwNP and AD were generated to evaluate early in its development the potential of PF-06817024 as a treatment for allergic diseases. The study was not designed nor powered for clinical efficacy as would be appropriate for Phase 2 studies. Nevertheless, exploring the activity of PF-06817024 in small cohorts enabled an efficient preliminary assessment of PD and clinical activity to inform future clinical development efforts. It remains to be determined whether prolonged treatment with PF-06817024 would be beneficial in patients with CRSwNP and AD.

In conclusion, PF-06817024 demonstrated modest clinical efficacy in reducing signs and symptoms of both CRSwNP and AD. Further investigation in appropriately powered Phase 2 studies would be necessary to define more fully the potential therapeutic benefit of PF-06817024 in CRSwNP and AD.

### Electronic supplementary material

Below is the link to the electronic supplementary material.

Supplementary Material 1

Supplementary Material 2

## Data Availability

Upon request, and subject to review, Pfizer will provide the data that support the findings of this study. Subject to certain criteria, conditions, and exceptions, Pfizer may also provide access to the related individual de-identified participant data. See https://www.pfizer.com/science/clinical-trials/trial-data-and-results for more information.

## Abbreviations

ACQ-5: 5-item version of the Asthma Control Questionnaire
AD: atopic dermatitis
ANCOVA: analysis of covariance
BMI: body mass index
BSA: body surface area
CCL17: CC motif chemokine ligand 17
CCL26: CC motif chemokine ligand 26
CI: confidence interval
CRSwNP: chronic rhinosinusitis with nasal polyps
CT: computerized axial tomography
EASI: Eczema Area and Severity Index
EASI50: ≥ 50% improvement from baseline in Eczema Area and Severity Index
EASI75: ≥ 75% improvement from baseline in Eczema Area and Severity Index
EASI90: ≥ 90% improvement from baseline in Eczema Area and Severity Index
FEV_1_: forced expiratory volume in 1 s
GPP: Good Publication Practice
hsCRP: high-sensitivity C-reactive protein
IGA: investigator global assessment
IgE: immunoglobulin E
IL: interleukin
ILC2: type 2 innate lymphoid cells
IV: intravenous
LSM: least squares mean
MMRM: mixed model for repeated measures
NPS: nasal polyp score
PD: pharmacodynamics
PK: pharmacokinetics
PRO: patient-reported outcome
SCORAD: Scoring Atopic Dermatitis
SD: standard deviation
SNOT-22: 22-item Sino-nasal Outcome Test
ST2: suppression of tumorigenicity 2
T_H_2: T helper 2
UPSIT: University of Pennsylvania Smell Identification Test
