# Supplementary material for: Exploratory pharmacodynamics and efficacy of PF-06817024 in a Phase 1 study of patients with chronic rhinosinusitis and atopic dermatitis
Source: Allergy Asthma Clin Immunol. 2024 Aug 30;20:46. doi: 10.1186/s13223-024-00894-8 (PMC11365161; doi:10.1186/s13223-024-00894-8)
Supplement: Supplementary file 1 — Supplementary Material 1 [file 13223_2024_894_MOESM1_ESM.docx]

# Supplemental Information

## Supplemental Results

- 1. **AD**
     1. **Efficacy endpoints – Day 113**

There were greater percentage decreases from baseline in investigator global assessment (IGA) scores in the PF‑06817024 group, compared with placebo, with a higher proportion of patients achieving an IGA response of ‘clear’ or ‘almost clear’ and ≥2 points improvement from baseline in the PF‑06817024 group. A greater percentage reduction from baseline in scoring atopic dermatitis (SCORAD) and change from baseline in body surface area (BSA) scores were also observed in the PF-06817024 group, compared with placebo. Decreases in Eczema Area and Severity Index (EASI), IGA, and SCORAD scores in patients with atopic dermatitis (AD) indicated improvements in AD severity, while reductions in BSA scores indicated a reduced extent of skin affiliated with AD.

- - 1. **PROs**

Greater percentage decreases from baseline in Patient Global Assessment and pruritus numerical rating scale (NRS), and change from baseline in POEM scores were observed in the PF-06817024 group during the treatment period (Day 2–113) and follow-up visits (Day 114–337), compared with placebo, which indicated lower severity and frequency of AD-induced itching, and improved eczema symptoms. Similar hospital and anxiety depression scale (HADS) and dermatology life quality index (DLQI) scores were observed at Day 113 in both the PF‑06817024 group and placebo group. In patients with comorbid asthma, decreases from baseline in the 5-item version of the Asthma Control Questionnaire (ACQ-5) scores were observed in the PF-06817024 group, compared with placebo, at Day 113, indicating improved asthma control.

For efficacy and patient-reported outcome (PRO) endpoints, data from Day 113 are shown in Table S1.

^
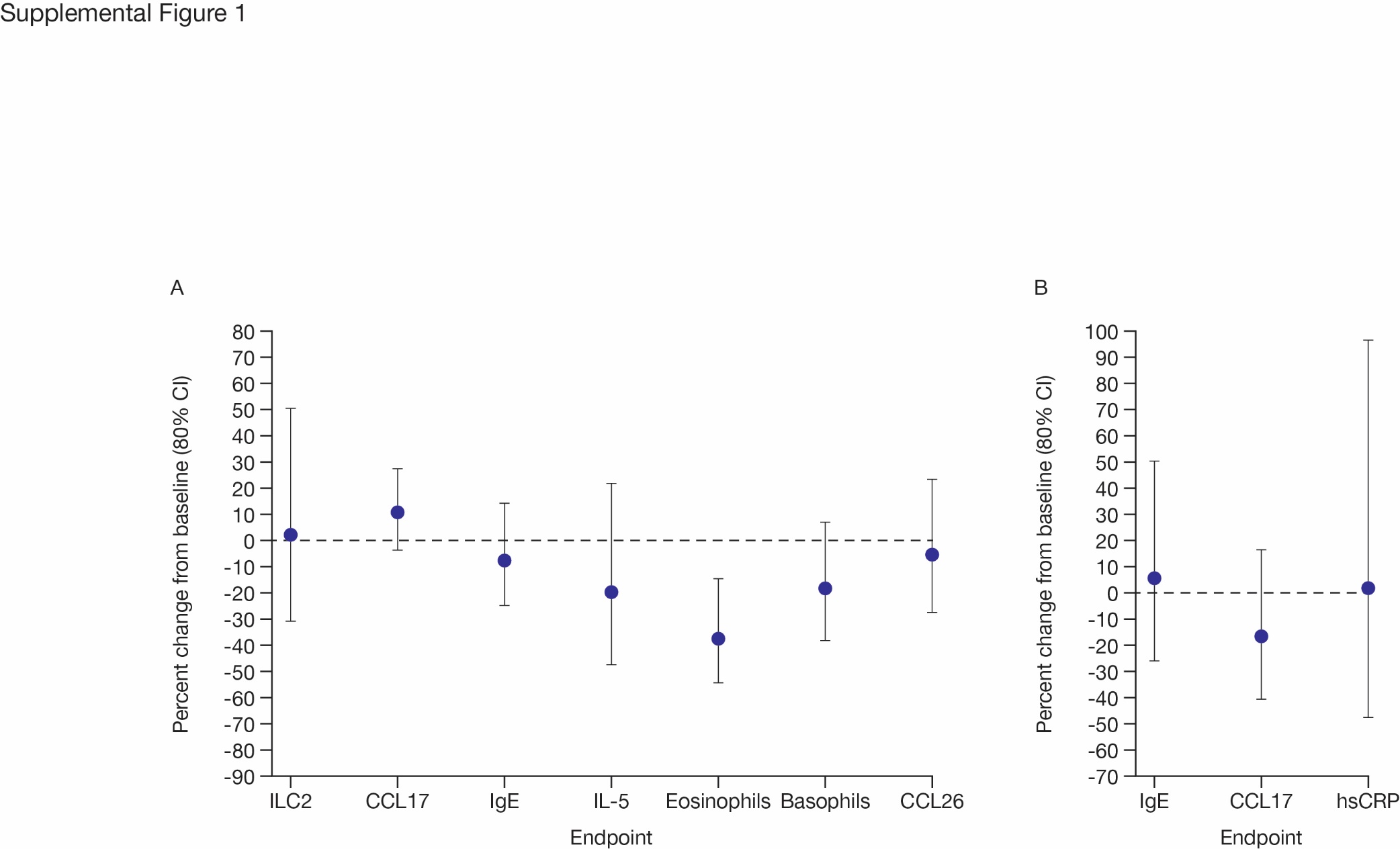
^**Fig. S1** Percentage change from baseline in biomarker endpoints (placebo-corrected). **A**, patients with CRSwNP at Day 61^a^ and **B**, patients with AD at Day 113^b^

Note: baseline is defined as the average measurement prior to the first dosing. Circles represented LSM of percentage change from baseline compared with placebo. The estimate and CI were calculated based on ANCOVA analysis with independent variable of treatment groups and baseline result. Bars represent 80% CI

^a^n=11 for all measurements in the PF-06817024 group; n=8 for ILC2, eosinophils, and basophils in the placebo group; n=9 for CCL17, IgE, IL-5, and CCL26 in the placebo group

^b^n=13 for IgE in the PF-06817024 group; n=14 for CCL17 and hsCRP in the PF-06817024 group; n=5 for all measurements in the placebo group

AD, atopic dermatitis; ANCOVA, analysis of covariance; CI, confidence interval; CRSwNP, chronic rhinosinusitis with nasal polyps; CCL17, chemokine (C-C motif) ligand 17; CCL26, chemokine (C-C motif) ligand 26; hsCRP, high-sensitivity C-reactive protein; IgE, immunoglobulin E; IL-5, interleukin-5; ILC2, type 2 innate lymphoid cells; LSM, least square means

**Fig. S2.** Individual EASI responses in AD at all timepoints. Percentage change from baseline in EASI by treatment group and EASI response


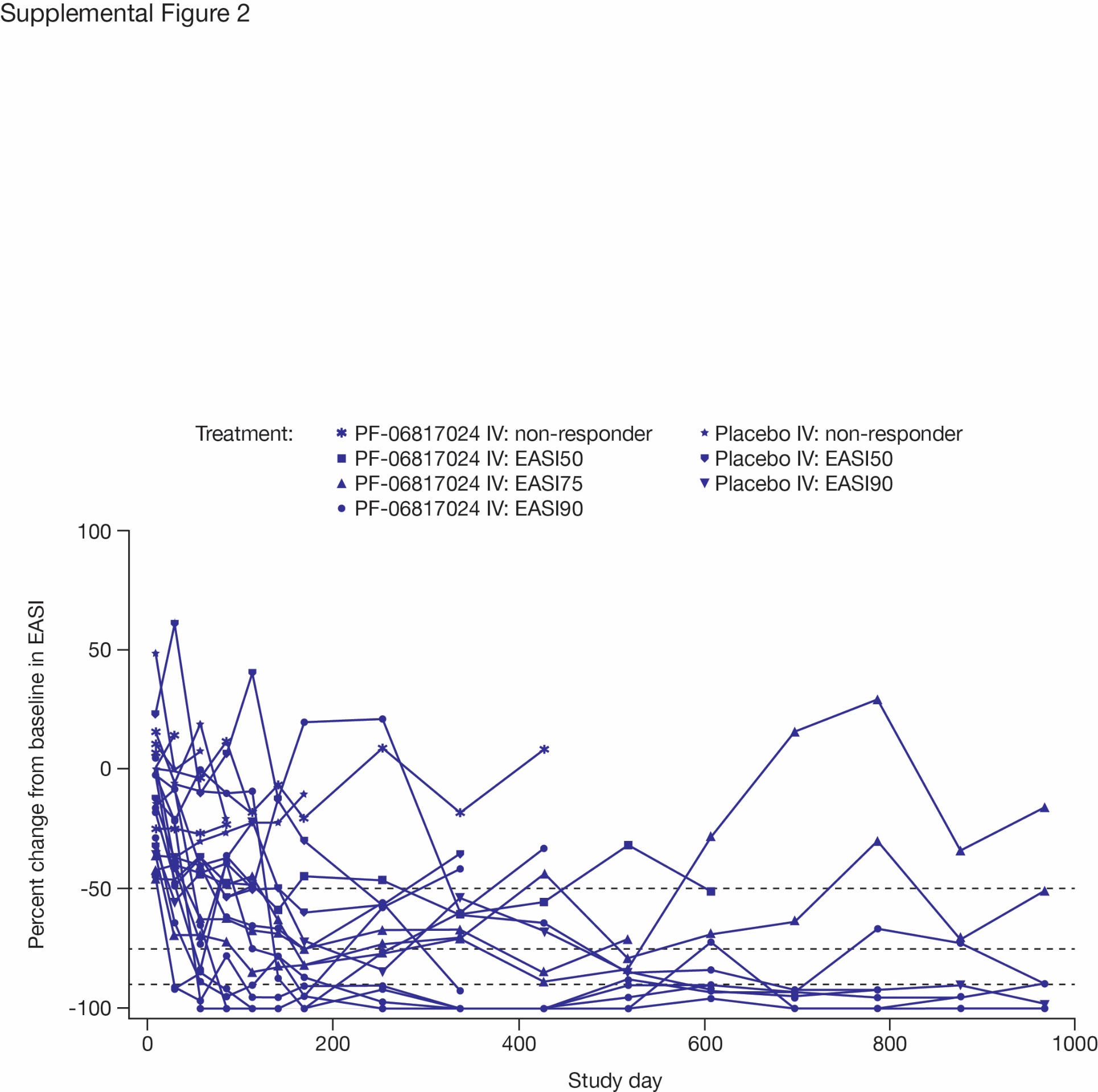


Note: baseline is defined as the last measurement prior to the first dosing

AD, atopic dermatitis; EASI, Eczema Area and Severity Index; EASI50, ≥50% improvement from baseline in Eczema Area and Severity Index; EASI75, ≥75% improvement in Eczema Area and Severity Index; EASI90, ≥90% improvement in Eczema Area and Severity Index; IV, intravenous

**Fig. S3.** Individual EASI responses in AD up to Day 113. Percentage change from baseline in EASI in individual participants by treatment group and EASI response


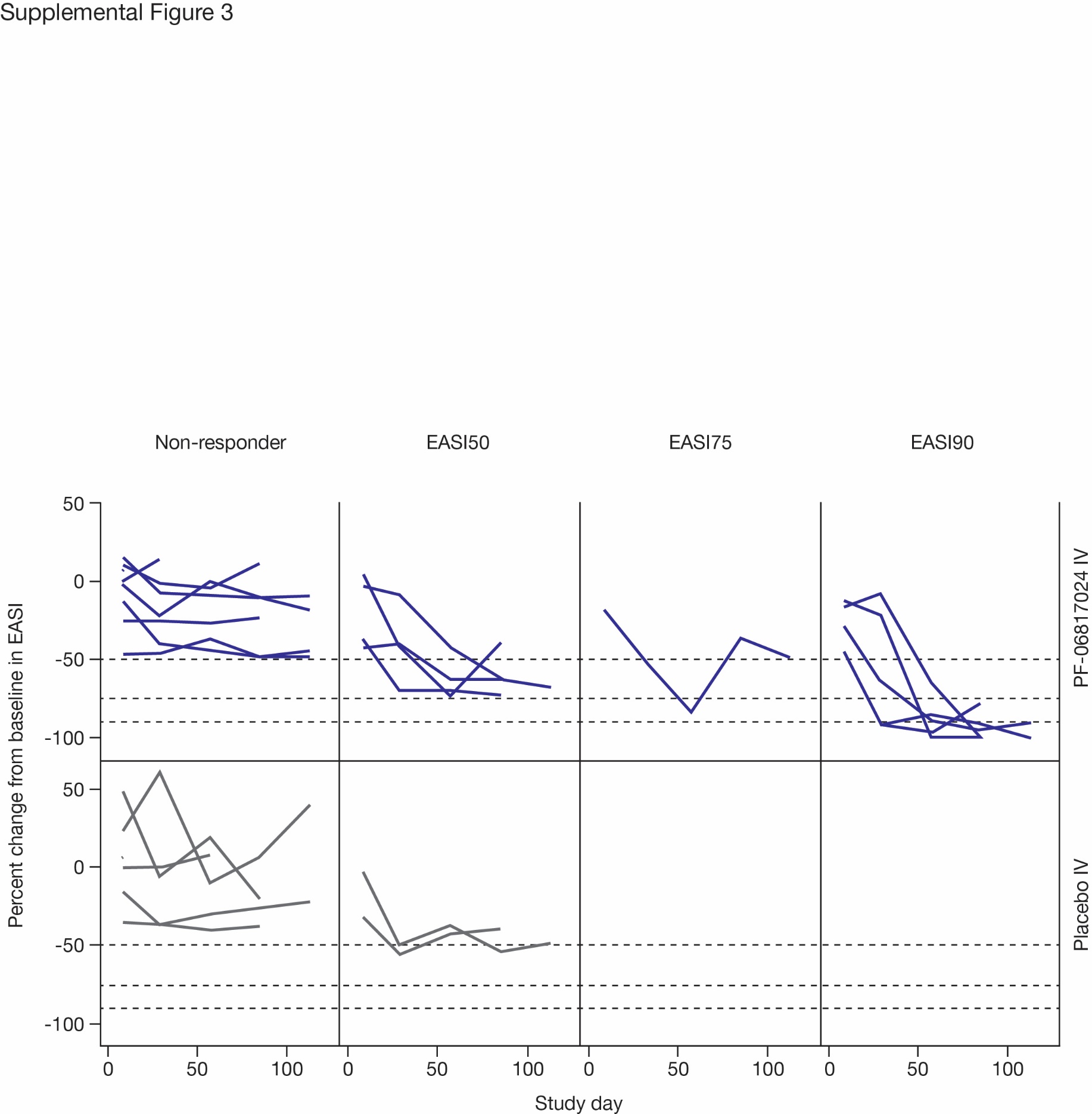


Note: baseline is defined as the last measurement prior to the first dosing

Two participants had missing data; one participant had only one result = 0 on Day 8, and one participant only collected early termination result

AD, atopic dermatitis; EASI, Eczema Area and Severity Index; EASI50, ≥50% improvement from baseline in Eczema Area and Severity Index; EASI75, ≥75% improvement in Eczema Area and Severity Index; EASI90, ≥90% improvement in Eczema Area and Severity Index; IV, intravenous
