# Supplementary material for: Exploratory pharmacodynamics and efficacy of PF-06817024 in a Phase 1 study of patients with chronic rhinosinusitis and atopic dermatitis
Source: Allergy Asthma Clin Immunol. 2024 Aug 30;20:46. doi: 10.1186/s13223-024-00894-8 (PMC11365161; doi:10.1186/s13223-024-00894-8)
Supplement: Supplementary file 2 — Supplementary Material 2 [file 13223_2024_894_MOESM2_ESM.docx]

**Table S1** Effect of PF-06817024 on efficacy and PRO endpoints at Day 113 in patients with AD

|  | **PF-06817024^d^** | **Placebo^d^** |
| --- | --- | --- |
| IGA; mean (SD)^a,b^ | -29.444 (36.7135) | -20.833 (25.0000) |
| SCORAD; mean (SD)^a,b^ | -40.678 (34.2600) | -31.131 (17.4811) |
| BSA; mean (SD)^b,c^ | -10.13 (12.110) | -19.40 (24.614) |
| PtGA; mean (SD)^a,b^ | -30.000 (31.1486) | -6.250 (12.5000) |
| Pruritus NRS; mean (SD)^a,b^ | -45.148 (38.4798) | 60.437 (66.9575) |
| POEM; mean (SD)^b,c^ | -10.2 (7.61) | -2.0 (7.16) |
| HADS; mean (SD)^a,b^ | -20.5 (52.48) | 12.7 (50.35) |
| DLQI; mean (SD)^a,b^ | -15.7 (121.26) | -5.3 (53.83) |
| ACQ-5; mean (SD)^b,c^ | -5.8 (8.84) | NE |

Note: baseline is defined as the last measurement prior to the first dosing

^a^Percentage change from baseline

^b^Decrease indicates improvement

^c^Change from baseline

^d^n=15 for IGA, SCORAD, BSA, PtGA, Pruritis NRS, and POEM in the PF-06817024 group; n=4 for IGA, SCORAD, BSA, PtGA, Pruritis NRS, and POEM in the placebo group; n=12 for HADS in the PF-06817024 group; n=5 for HADS in the placebo group; n=14 for DLQI in the PF-06817024; n=5 for DLQI in the placebo group; n=5 for ACQ-5 in the PF-06817024 group; n=0 for ACQ-5 in the placebo group

ACQ-5, 5-item version of the Asthma Control Questionnaire; AD, atopic dermatitis; BSA, body surface area; DLQI, Dermatology Life Quality Index; HADS, Hospital and Anxiety Depression Scale; IGA, Investigator Global Assessment; NE, not evaluated; NRS, numerical rating scale; POEM, Patient-Oriented Eczema Measure; PRO, patient-reported outcome; PtGA, Patient Global Assessment; SCORAD, Scoring Atopic Dermatitis; SD, standard deviation
